# Ablation of SGK1 Impairs Endothelial Cell Migration and Tube Formation Leading to Decreased Neo-Angiogenesis Following Myocardial Infarction 

**DOI:** 10.1371/journal.pone.0080268

**Published:** 2013-11-12

**Authors:** Elham Zarrinpashneh, Tommaso Poggioli, Padmini Sarathchandra, Jonas Lexow, Laurent Monassier, Cesare Terracciano, Florian Lang, Federico Damilano, Jessica Q. Zhou, Anthony Rosenzweig, Nadia Rosenthal, Maria Paola Santini

**Affiliations:** 1 Heart Science Centre, National Heart and Lung Institute, Imperial College London, Harefield, United Kingdom; 2 Laboratoire de Neurobiologie et Pharmacologie cardiovasculaire, Strasbourg, France; 3 Physiologisches Institut der Universität Tübingen, Tübingen, Germany; 4 Cardiovascular Division, Beth Israel Deaconess Medical Center and Harvard Medical School, Boston, Massachusetts, United States of America; 5 Australian Regenerative Medicine Institute, European Molecular Biology Laboratory Australia/Monash University, Melbourne, Australia; University College London, United Kingdom

## Abstract

Serum and glucocorticoid inducible kinase 1 (SGK1) plays a pivotal role in early angiogenesis during embryonic development. In this study, we sought to define the SGK1 downstream signalling pathways in the adult heart and to elucidate their role in cardiac neo-angiogenesis and wound healing after myocardial ischemia. To this end, we employed a viable SGK1 knockout mouse model generated in a 129/SvJ background. Ablation of SGK1 in these mice caused a significant decrease in phosphorylation of SGK1 target protein NDRG1, which correlated with alterations in NF-κB signalling and expression of its downstream target protein, VEGF-A. Disruption of these signalling pathways was accompanied by smaller heart and body size. Moreover, the lack of SGK1 led to defective endothelial cell (ECs) migration and tube formation *in vitro*, and increased scarring with decreased angiogenesis *in vivo* after myocardial infarct. This study underscores the importance of SGK1 signalling in cardiac neo-angiogenesis and wound healing after an ischemic insult *in vivo.*

## Introduction

Neo-angiogenesis, the process of new blood vessel formation from the pre-existing network of capillaries, is a response to many physiological and pathological conditions such as myocardial ischemia, diabetes and cancer [1,2]. In the heart, activation of neo-angiogenesis around the ischemic area helps to supply oxygen and nutrients thereby decreasing cell death and scar formation [1,2]. During embryonic development, serum and glucocorticoid inducible kinase 1 (SGK1) is reported to be an important player in early angiogenesis [3]. SGK1 is a serine-threonine protein kinase first cloned from rat mammary tumours in response to glucocorticoid stimulation [4]. SGK1 belongs to the AGC kinase subfamily, acting downstream of insulin and growth signalling pathways in response to phosphorylation by PIP3-dependent protein kinase-1 (PDK1) [5]. Two additional distinct SGK isoforms have been identified (SGK2 and SGK3). All SGK isoforms share similar target specificities with other AGC family members such as Akt (PKB), RSK, and S6K [6]. 

N-Myc downstream regulated gene-1 (NDRG1, Cap 43, Drg1) is the specific physiological substrate for SGK1 [7]. NDRG1 expression is regulated by oncogenes (N-myc and C-myc) and tumour suppressors (p53, VHL, and PTEN) [8]. Over-expression of NDRG1 has been shown to suppress tumour growth through modification of angiogenesis [9]. Both over-expression and phosphorylation of NDRG1 by SGK1 have been reported to inhibit the nuclear factor kappa B (NF-κB) pathway and decrease expression of angiogenic genes such as CXC chemokines and vascular endothelial growth factor-A (VEGF-A) [9,10].

In previous analyses, we found that SGK1-/- mice, generated as 129/SvJ background are viable with no significant cardiac phenotype [11]. Nevertheless, when backcrossed for more than five generations in C57BL/6 background they showed embryonic lethality due to an early cardiovascular defect at E10.5-E11.5 [3]. Based on these data, we investigated the role of SGK1 and NDRG1 phosphorylation in cardiac neo-angiogenesis and wound healing in adult SGK1 null mice on a 129/SvJ mouse background. We show that inactivation of SGK1 caused a dramatic decrease in NDRG1 phosphorylation in the heart and endothelial cells (ECs). NDRG1 modulation was associated with degradation of NF-κB inhibitory proteins and increased in VEGF-A protein expression. This disrupted signalling resulted in defective migratory and *in vitro* tube formation capacity of SGK1-/- ECs compared to wild-type (WT) ECs. Importantly*, in vivo* analyses showed that SGK1 deletion worsened scar formation a month after coronary artery ligation due to lower density of vessels per cardiomyocyte around the scar area compared to WT mice. Our results elucidate the role of SGK1 signalling in the regulation of angiogenesis and wound healing in the adult heart, an effect involving phosphorylation of its downstream substrate NDRG1. 

## Materials and Methods

### Mice

All animal studies and breeding protocols were performed in compliance with international (Directive 2010/63/EU of the European Parliament) and national (UK Home Office, Act 1986) regulations. Imperial College board Committee granted internal ethical approval. All animals were examined daily for development of any adverse signs and symptoms indicating pain, distress or discomfort. Any animal giving cause for concern was weighed and monitored and if there was body weight loss of more than 20% and/or significantly laboured breathing as well as the following clinical signs: piloerection, hunched posture, reduced mobility, pallor, ocular or nasal discharge, diarrhoea was humanely culled as described below. In all experimental procedures mice were anaesthetised with inhaled Isoflurane (1.5-2.5%) and 1.5 ml/min O_2_. Adequacy of anaesthesia was monitored by foot pinch before incision. For tissue extraction and primary cell isolation, mice were euthanized by cervical dislocation after being anesthetised with 4% Isoflurane (National Veterinary Services, NVS, UK). After surgery, animals were allowed to recover with free access to food and water. Injection of analgesia (e.g. buprenorphine) was performed as required post-operatively. SGK1-/- mice were genotyped as previously described [11]. Male mice were used for physiological studies and isolation of cardiomyocytes and female mice were used for isolation of ECs.

### Materials

Antibodies: pan SGK (≠3272), SGK2 (≠5595), SGK3 (≠8156), p-NDRG1-Thr346 (≠5482), NF-κB_2_/p100 (≠4882), GAPDH (≠2118) were purchased from Cell Signalling; total NDRG1 from university of Dundee (DSTT); VEGF-A (≠sc-507), and inhibitor of kappa-B alpha (IκBα) (≠sc-56710) antibody from Santacruz; Isolectin beta-4 (ILB4) (≠L2140) and Wheat-germ agglutinin (WGA) (≠L4895) from Sigma; Matrigel from BD Biosciences (≠734-0269); Proliferation kit from Roche (≠11 810 740 001); CytoSelect migration assay (≠CBA-106) from Cell Biolab and di-8-ANEPPS from Molecular Probes.

### Echocardiography

2-3 month old WT and SGK1-/- mice were analysed under anaesthesia (2.5% Isoflurane, 1.5 ml/min O_2_). Short-axis view trans-thoracic echocardiography (ECHO) was performed on shaved mice at the height of the papillary muscles. The operator was blinded at the time of measurement to genotype of each mouse analysed. Ejection fraction (%EF) was determined in 2D and M-mode, fractional shortening (%FS) was measured in M-mode by a Sonos 5500 (Philips) equipped with a 15MHz transducer. 

### Coronary artery ligation

Left coronary artery ligation was performed on 3 month old mice as previously described [12]. In brief, mice were anaesthetized with 1.5% Isoflurane and the chest cavity was opened in the left fourth intercostal space. The heart was exposed and the left anterior descending coronary artery (LAD) was ligated with an 8.0 non-absorbable suture (Ethicon) below the left atrium to produce an infarct size of about 40%. Mice were sacrificed one month after ligation and samples were collected for immunohistochemistry, protein and RNA analysis.

### Immunohistological analysis

Hearts were harvested, washed in PBS and fixed in 4% paraformaldehyde. The fixed heart samples were then embedded in paraffin and 5 µm microtome sections were used for different staining after being deparaffinised and boiled for 10 minutes in 10 mM sodium citrate (pH=6). Microvessels were stained with endothelial cell marker ILB4 (Biotinylated) at a 100 fold dilution and cardiomyocytes were stained with the marker Wheat-germ agglutinin (WGA), FITC conjugated, at a concentration of 5µg/ml. Microvessels and cardiomyocytes were counted in 10 defined microscope fields. To quantify the scar area after coronary artery ligation, heart sections were stained with picro-sirius red for collagen deposition. Three different sections at the start, mid and the end of scar were used for staining and the percentage of scar size was reported as the mean value of epicardial and endocardial scar in each section using NIS-element AR3.0 software.

### Immunoblotting

Western blot analysis performed on tissue and/or cell extracts as previously described [13]. In brief 40-50µg protein samples were loaded to SDS-gel followed by electrotransfer to a nitrocellulose membrane. Primary antibodies were applied at a 1000 fold dilution for commercial antibodies and of 0.1 µg/ml concentration for Total NDRG1 (Dundee) in TBS/Tween overnight at 4°C. Protein detection was performed using horseradish peroxidase conjugated secondary antibodies and the enhanced chemiluminescence reagent.

Bands were quantified with ImageJ, a Java-based image analysis package widely used for measurement of density profiles, peak heights as well as peak intensity (average OD of the band, INT) or volume (average OD of the band times its area, INT*mm^2^) of the band of the expected molecular weight. Briefly, after scanning the western blot films, the images were saved as jpg format. After opening the image in the Image J program, the area around each band was specified by rectangular selection in the program tools. The program then plots the same rectangular around all the other bands and quantifies the intensity of each band. The intensity of each band was then normalised with the intensity of corresponding loading controls.

### Real Time PCR

Total RNA was extracted and quantitative real-time reverse-transcribed polymerase chain reaction was performed as described previously [14] with off-the-shelf Taqman probes (Applied Biosystems). Analysis was performed with the ΔΔCt method with an 18S probe for normalization.

### Primary cell preparation

Cardiomyocytes were isolated from 2-3 month old male mice after standard enzymatic digestion as previously described [15]. Cardiomyocytes were then stained with the membrane binding dye di-8-ANEPPS (10µM) for 10 minutes in the dark. Confocal image stacks were obtained using the Zeiss LSM 510 microscope with 40x objective lens using Argon 488 laser. The images were analysed to calculate cell volume as previously described [16].

ECs were isolated from 2-3 month old mice. After euthanasia hearts were excised and minced in cold PBS. Single cell isolation was achieved after digestion of hearts in collagenase IV (Roche) for one hour at 37°C. Cell debris was discarded through a cell strainer (100µm) and cardiomyocytes were removed after first centrifugation at 20g. The remaining cells were washed by centrifugation at 300g several times with PBS/EDTA/BSA buffer (PEB buffer) containing phosphate buffer saline (PBS) 1X, pH 7.2, 0.5% bovine serum albumin (BSA) and 2 mM EDTA. Cells were then labelled with EC marker CD146 (Miltenyi Biotec ≠130-092-007) and selected through magnetic cell separation (MACS) columns as recommended by manufacture’s experimental procedure (Milteny Biotec, Inc.). ECs were cultured in DMEM supplemented with 10% FCS, endothelial cell growth factors (ECGS) (Sigma) and heparin in 1% fibronectin pre-coated culture dishes for 1 week. 

### EC proliferation analysis

ECs were cultured on chamber slides to reach 50% confluence. Proliferation of ECs was measured using *In Situ* Cell proliferation kit (Roche) according to the manufacturer. In brief ECs were incubated with a thymidine analogue, 5-bromo-2`-deoxyuridine (BrdU) at the concentration of 10 µM for 24h. At the end of the incubation time, cells were fixed with a mixture of ethanol/glycine (50 mM, pH 2.0), washed with PBS and stained with anti-BrdU-FLUOS antibody for 45 minutes followed by DAPI staining for 5 minutes. The number of BrdU positive cells was evaluated by Zeiss fluorescent microscopy and normalized by the total number of cell nuclei stained with DAPI.

### EC migration analysis

Primary ECs from mice heart were serum starved for 12h and subsequently seeded at 1-2x10^4^ cells per well in a serum-free media into the upper chamber of CytoSelect™ 96-well cell migration (Cell Biolabs) covering the lower chamber with a polycarbonate membrane with a diameter of 8 µm. Cells were allowed to migrate toward 10% FCS for 24h. The migratory cells on the bottom of the membrane were then detached and quantified using CyQuant GR fluorescent dye at 480nm/520 nm wavelength using fluorescent plate reader as described by manufacturer (Cell Biolab). 

### EC tube formation analysis

1-2x10^4^ primary ECs were seeded on Martigel (BD Biosciences) coated 12 well plate culture dishes. Tube formation was visualized at 10X magnification with Zeiss light microscopy after 7 and 24h. In a subset of experiments, VEGF-A was neutralized by adding VEGF-A antibody (2ug/ml) and control rabbit IgG to the media as previously described [17]. Tube formation was quantified by tracing along the tubes using NeuronJ software (an ImageJ plugin). The number of traces per 8 bit jpeg images was quantified from 10 different culture dishes. The significance of the results was assessed using a Student-*t* test.

### Kinexus Proteomic Analysis

Three WT and SGK1-/- hearts were lysed in non-denaturing buffer purchased from Kinexus Bioinformatics Corporation (Vancouver, Canada). Samples were delivered to Kinexus in dry ice and subsequently labelled with fluorescent dye. Labelled samples were run on a Kinex^TM^ 850-KAM antibody microarray chip containing 850 different antibodies. Detected fluorescent samples were quantified and the differences between WT and KO analysed as Z-ratio (KO/WT fluorescent intensity). 

### Adenovirus infection

Three months old WT and KO mice were used to extract endothelial cells as described above. Isolated cells were maintained in DMEM supplemented with 10% FCS, endothelial cell growth factors (ECGS) (Sigma) and heparin in 1% fibronectin pre-coated culture dishes for 1 week. Cells were then trypsinized and plated 50-60% confluency in EC media for 12 hours, prior infection with an adenovirus expressing the constitutively active form of SGK1 and a control adenovirus expressing beta-Galactosidase (βGal) [18]. Cells were infected for 12 hours in DMEM containing 10% FBS followed by plating on matrigel coated dishes as described above. Tube formation was assessed 7 hours after plating. 

### Statistical analysis

GraphPad Prism (version 5) was used to perform statistical analysis. Student *t* -test analysis was performed for normally distributed data. ANOVA was used as indicated in Figure legends. Results were presented as the average± SE. *p<0.05, **p<0.01, ***p<0.001

## Results

### SGK1-/- mice display a cardiac phenotype

Generation of SGK1-/- mice has been described previously [11]. In brief, exons 4-11, which encode the SGK1 kinase domain, were constitutively deleted in the germ line using Cre-lox technology. The mice were fertile and showed no significant phenotype apart from their smaller body weight and heart weight (Table 1). Echocardiography analyses revealed that cardiac systolic and diastolic functions defined by %EF, %FS and E/A ratio respectively were comparable between WT and SGK1-/- mice. However, a significantly slower heart rate was observed in SGK1-/- mice (Table 1). 

**Table 1:** Cardiac phenotype of SGK1-/- mice.

	**WT**	**SGK1-/-**
**Morphologic:**	***(n=8)***	***(n=7)***
HW (mg)	190±3	120±5*
HW/BW	5.5±0.1	5.1±0.2
BW (g)	34±1	23.4±0.9*
**Echocardiographic:**	***(n=8)***	***(n=7)***
LVEDD (mm)	4.33±0.0	4.17±0.0
LVESD (mm)	2.91±0.1	2.64±0.1
EF%	67±3	72±2
FS%	33±2	36±1.6
HR	415±2	319±13**
E/A	1.5±0.2	1.7±0.14

Mice were weighed at 8 weeks and dissected wet heart weight measured. 2-3 month old male mice were used for functional analysis of the heart by measuring the percentage of ejection fraction (%EF) and fractional shortening (%FS). n, number of mice, BW, body weight, HW, heart weight; HW/BW, ratio of HW to BW; LVEDD, left ventricular end diastolic diameter; LVESD, left ventricular end systolic diameter; HR, heart rate. E/A, maximal speed of early to late mitral filling ratio. Student t-test has been used to calculate significance and * p<0.05; **p<0.01.

**Figure 1:**
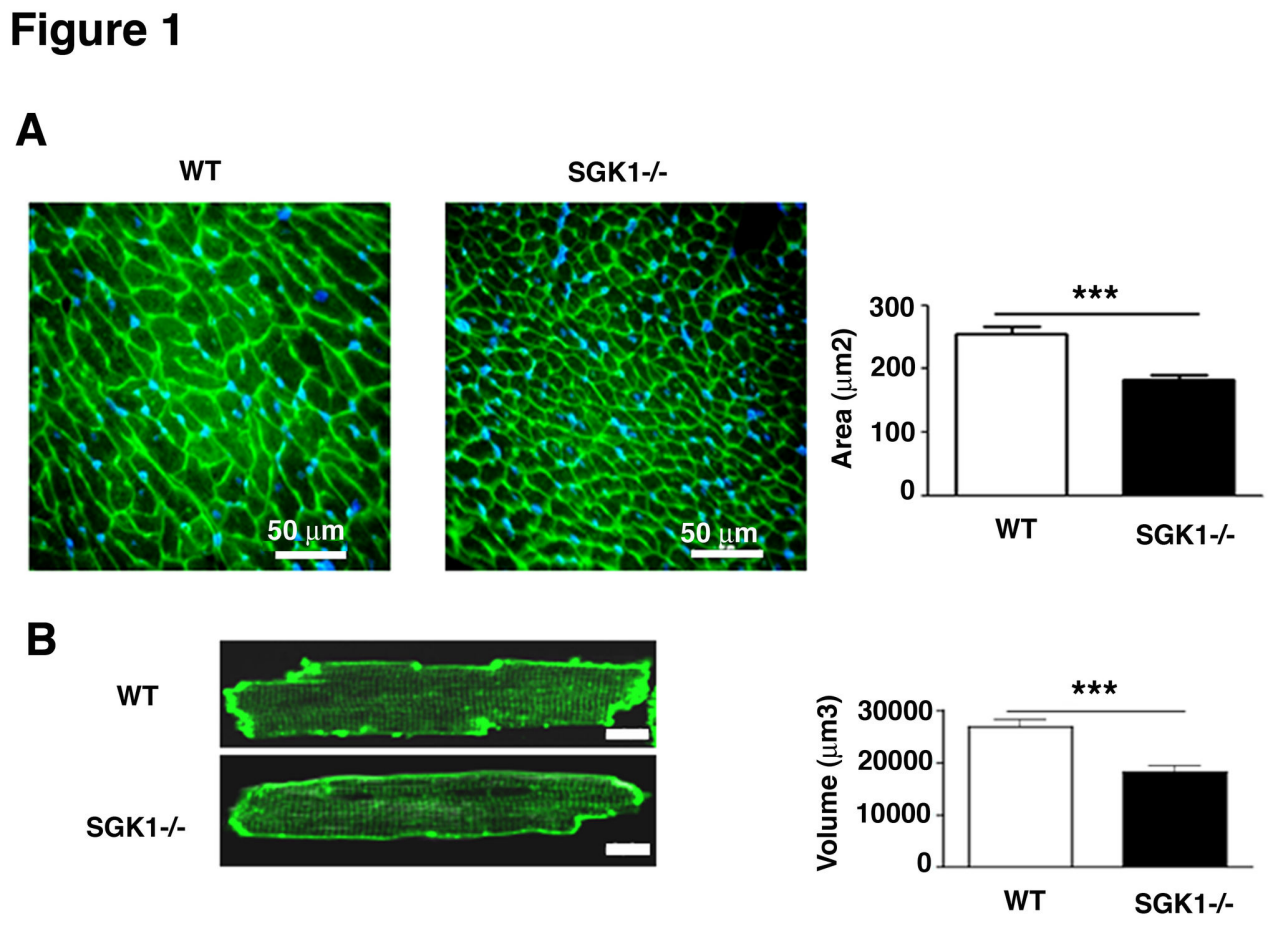
Lack of SGK1 correlated with reduced cardiomyocyte size. (A) Cross sectional area in heart sections from WT and SGK1-/- mice. Area was measured in ≥10 distinct microscope field for each slide. Four hearts were analysed for each slide in both WT and SGK1-/- mice. Scale bar represents 50 µm. (B) Confocal images of isolated adult cardiomyocytes form WT and SGK1-/- mice The data represent the average of three independent experiments and are reported as Mean ± SE. Cell volume was measured using a custom-written macro in ImageJ. Scale bars represent 5µm. Statistical analysis has been performed as described in Materials and Methods using Student t-test; *** indicates p<0.001.

Decreased heart weight observed in SGK1-/- was accompanied by reduced cardiomyocyte size evaluated by cross-sectional area in WGA stained heart sections (Figure 1A) and by cell volume measured in isolated adult cardiomyocytes (Figure 1B). 

### Reduced NDRG1 phosphorylation in SGK1-/- hearts

**Figure 2:**
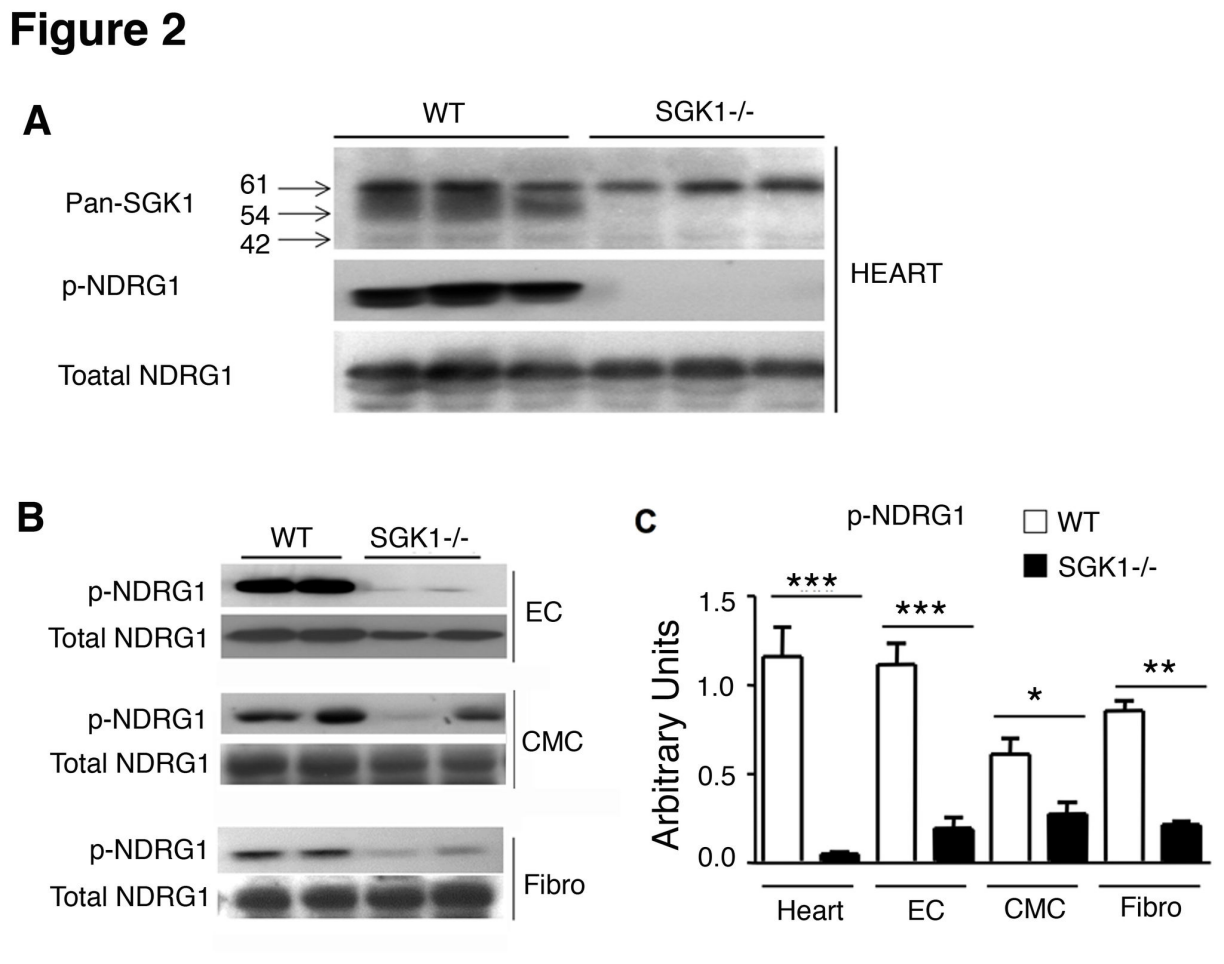
Downregulation of NDRG1 phosphorylation in SGK1-/- hearts. Western blot analysis of SGK, phospho-NDRG1 and total NDRG1 in the heart (A), primary endothelial cells (ECs), adult cardiomyocytes (CMC) and fibroblasts (Fibro) from WT and SGK1-/- mice (B). Quantification of band intensities was performed using ImageJ. The number of hearts analysed was ≥4 in each group (C). Significance has been measured by Student t-test has described in Materials and Methods and *** indicates p<0.001, ** p<0.01 and * p<0.05. The data are reported as Mean ± SE.

To investigate the effectiveness of SGK1 deletion, heart samples from WT and SGK1-/- mice were analysed for SGK protein expression by Western blotting. As shown in Figure 2A, the pan SGK antibody recognizes different SGK isoforms. However, the expression of the middle band at 54 KDa corresponding to SGK1 isoform was abolished in SGK1-/- mice. To further investigate the ablation of SGK1 in these animals, the phosphorylation state of NDRG1, a downstream target of SGK1 was examined. NDRG1 is specifically phosphorylated by SGK1 at residue threonine-346 [7]. Correspondingly, Western blot analysis of SGK1-/- whole heart samples showed a significant decrease in NDRG-1 phosphorylation at threonine-346 (Figure 2A). Decreased NDRG-1 phosphorylation was also observed in primary ECs, adult cardiomyocytes and fibroblasts isolated from SGK1-/- mice (Figure 2B and 2C). Importantly, the expression levels of the other isoforms SGK2 and SGK3 in the heart assessed by western blot did not show any significant difference between WT and SGK1-/- mice, ruling out any compensatory modifications in the level of these isoforms (Figure S1).

### Modification of NF-κB pathway in SGK1-/- hearts

**Figure 3:**
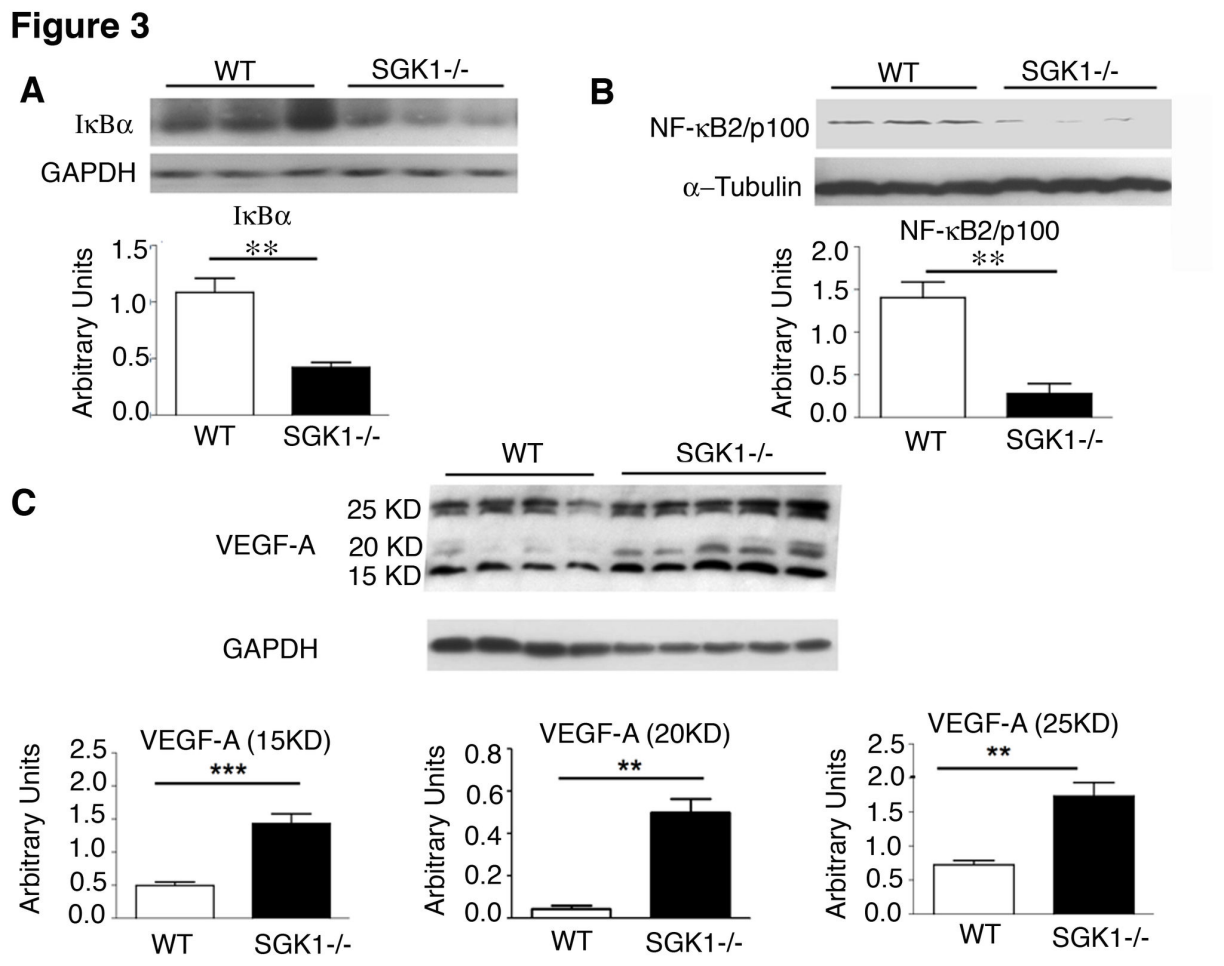
Modulation of NF-κB pathway and VEGF-A expression by SGK1. Western blot analysis of IκBα (A), NF-κB_2_/p100 (B) and VEGF-A (C) in WT and SGK1-/- hearts. GAPDH and α-Tubulin were used as loading controls. Quantification of band intensities for each represented blot was performed using ImageJ. The number of hearts analysed was ≥4 in each group and statistical analysis is performed by Student t-test. ** Indicates p<0.01, *** p<0.001. The data are reported as Mean ± SE.

To investigate NF-κB signalling, which is downstream of the SGK1/NDRG1 pathway, Western blot analysis was performed on WT and SGK1-/- heart samples for two well-known inhibitory components of NF-κB pathways: IκBα in the canonical and NF-κB_2_/p100 in the non-canonical pathway [19]. The expression level of VEGF-A was also assessed by Western blot as a transcriptional target of NF-κB [20]. As shown in Figures 3A and 3B, expression of both inhibitory proteins, IκBα and NF-κB_2_/p100, was downregulated in SGK1-/- heart samples. Decreases in these inhibitory components were accompanied by a significant increase in all VEGF-A isoforms (25, 20 and 15 KDa) in SGK1-/- heart samples (Figure 3C). Furthermore, extracts from cardiac cell types showed that endothelial cells are the main producers of VEGF in SGK1 KO hearts compared to cardiomyocytes, secreting similar levels of this factor (Figure S2A and S2B).

### Proteomic profile of SGK1-/- hearts

To investigate whether deletion of SGK1 was involved in regulating other downstream targets, we performed proteomic analyses by assessing the expression and phosphrylation profile of 850 different proteins using a Kinexus antibody microchip array (Figure S3). Our data showed that depletion of SGK1 is associated with differential regulation of proteins implicated in cell cycle initiation or progression such as Aurora A (1.5+/-0.41 fold induction compared to WT heart) [21], PAC1, regulator of mitotic and apoptotic signalling (3.6+/-1.9 fold induction compared to WT) [22,23] and CDK7 activator of transcription [24] (-4.7+/-2.7 fold reduction compared to WT). Interestingly, Btk, known to activate NFkB [25,26], is induced a 1.4+/-0.3 fold in SGK1-/- hearts compared to WT, further corroborating the data presented in this manuscript.

### Defective tube formation in SGK1-/- ECs

**Figure 4:**
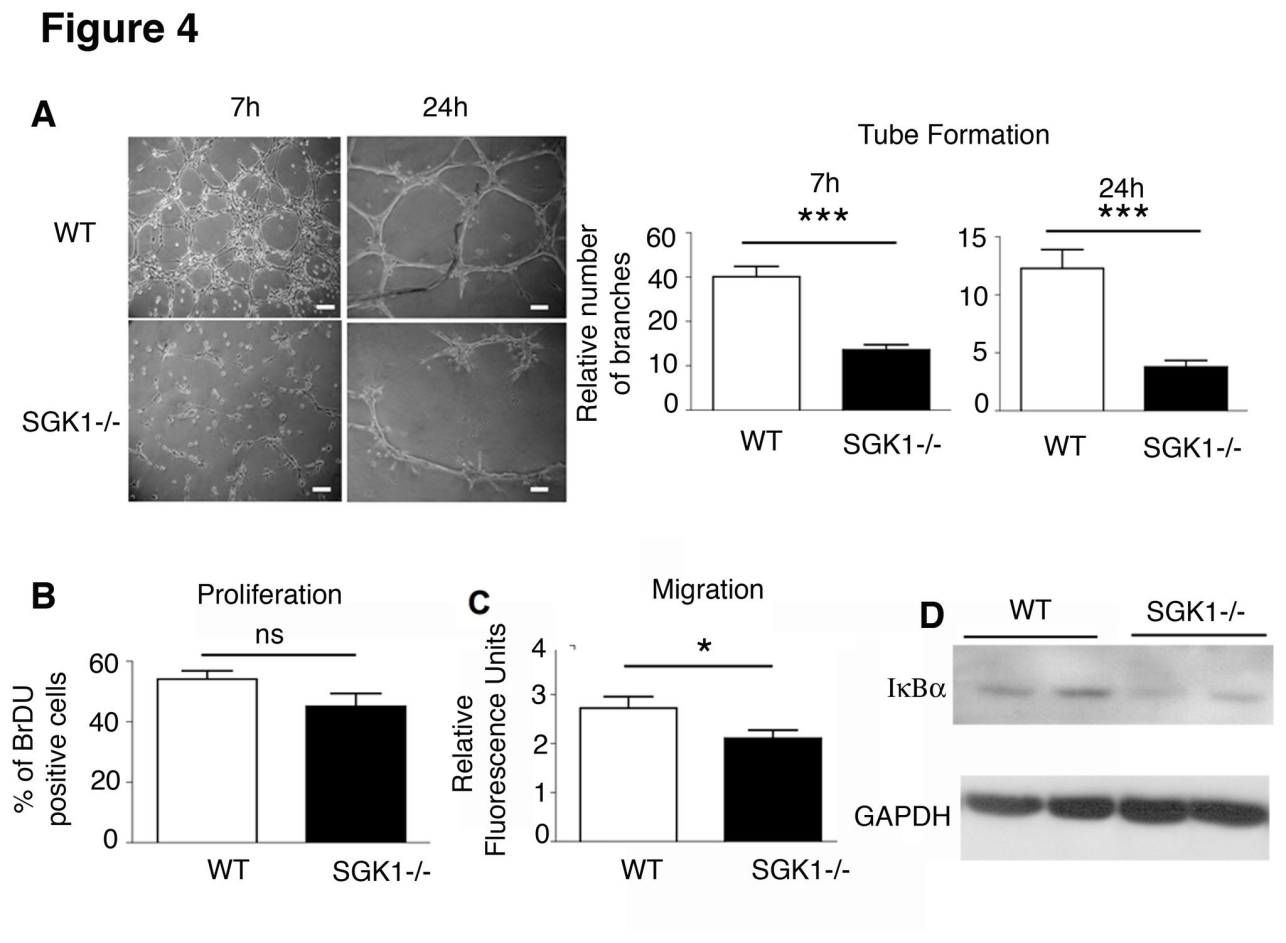
SGK1 affects EC phenotype. Primary ECs from WT and SGK1-/- hearts were assessed for tube formation capacity after culture on Matrigel for 7 and 24h. Tube ramification was quantified using Neuron J software. Scale bars represent 100 µm (A). Proliferation capacity of ECs measured 24h after incubation with BrdU (B). Endothelial cell migration capacity was measured using CytoSelect cell migration assay (C). NFkB signalling was measured by analysing the expression levels of the inhibitory protein IkBα. Blots were normalized for GAPDH. Two representative experiments are shown. Four hearts were used in both WT and SGK1-/- mice and statistical analysis has been performed by Student t-test as described in Materials and Methods. Ns indicates no significant; * p<0.05, ***p<0.001. The data are reported as Mean ± SE.

The role of SGK1 in EC tube formation was examined by culturing primary ECs isolated from WT and SGK1-/- mice on Matrigel-coated culture dishes. As shown in Figure 4A, ECs isolated from SGK1-/- mice formed fewer tube networks after 7 and 24 h, compared to WT cells. Importantly, re-expression of SGK1 by infection of ECs with an adenovirus encoding the constitutively active form of SGK1 (Figure S4A and S4C) re-established tube networks compared to KO ECs infected with control virus expressing βGal (Figure S4B). 

To investigate whether this phenotype was due to a defect in proliferative capacity of SGK1-/- ECs, we incubated the ECs with a thymidine analogue, 5-bromo-2`-deoxyuridine (BrdU) for 24h. Figure 4B presents the ratio of BrdU incorporated cells per total amount of cells stained with DAPI. No significant differences were observed between WT and SGK1-/- ECs, excluding altered proliferative capacity as an explanation for lower tube formation. Assessment of ECs migratory capacity showed a significantly higher migration level in ECs isolated from WT than from SGK1-/- hearts (Figure 4C), indicating that SGK1 signalling supports ECs migration. We further analysed the effect of VEGF-A on ECs tube formation by neutralizing VEGF-A in the media. Our results demonstrated that blocking VEGF-A partially improved SGK1-/- ECs capacity to form tubes (Figure S2C). To analyse whether ECs contribute to the signalling observed in the hearts, expression of NFκB inhibitory proteins was measured by western blot analysis. In Figure 4D, it is shown that IκBα expression in the canonical pathway is downregulated, although no differences were found in NF-κB_2_/p100 expression levels (communication from the authors). These data suggest that the canonical pathway of NFκB is in part regulating EC function.

### Modulation of SGK1 signalling in SGK1-/- hearts in response to myocardial ischemia

**Figure 5:**
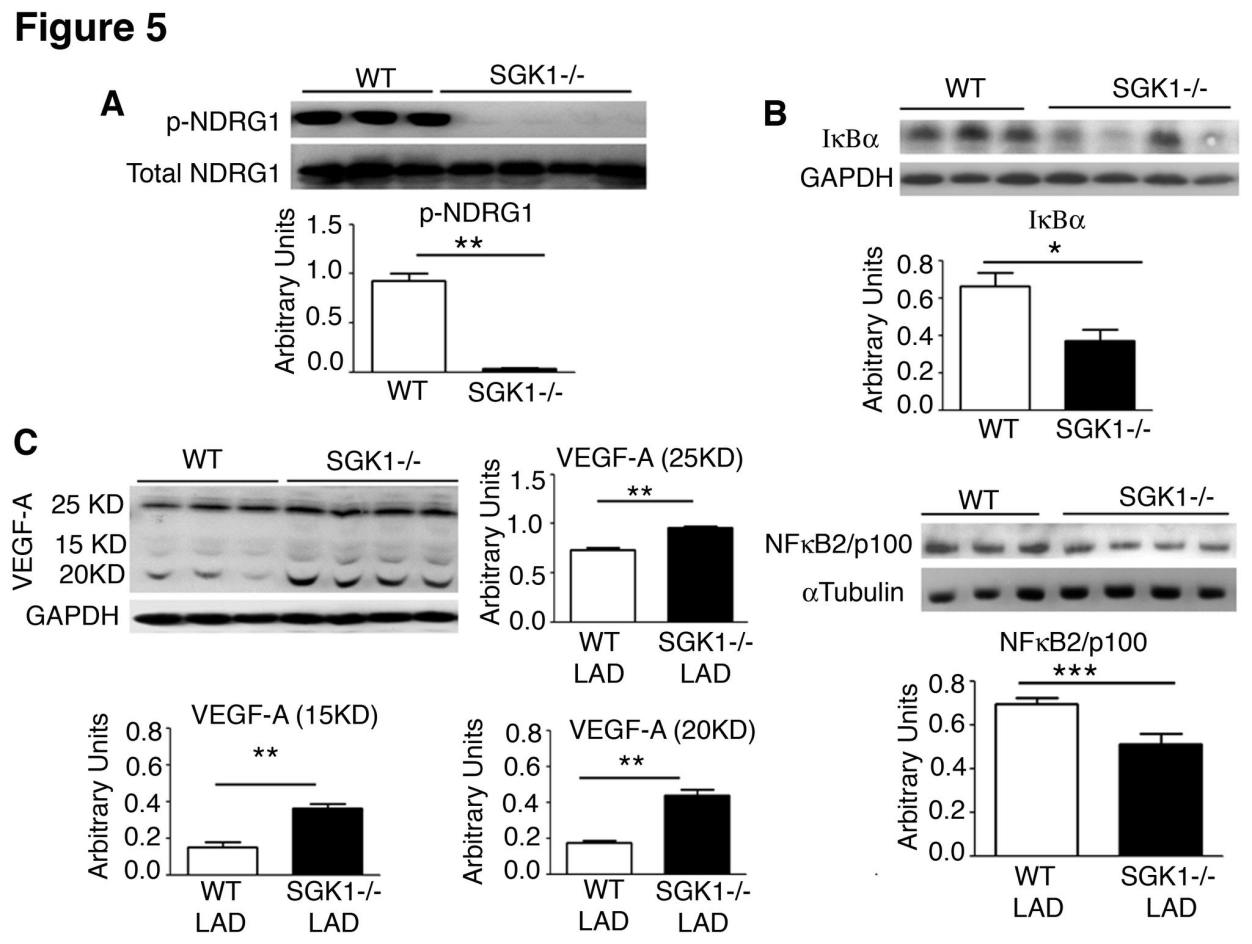
Disruption of SGK1 downstream signalling after coronary artery ligation. Western blot analysis of NDRG1 (A), IκBα, NF-κB_2_/p100 (B) and VEGF-A (C) of WT and SGK1-/- hearts after LAD. GAPDH and α-Tubulin were used as loading controls. Quantification of band intensities was performed using ImageJ. The number of hearts analysed was ≥4 in each group. Significance is calculated by Student t-test and * p<0.05, ** p<0.01, ***p<0.001. The data are reported as Mean ± SE.

To investigate the impact of defective SGK1 activity on cardiac recovery after ischemic insult, mice were subjected to left anterior descending coronary artery ligation (LAD). Assessment of SGK1 downstream pathways was performed by western blot analysis one month after coronary artery ligation to explore whether ischemic insult modulated the signalling observed in physiological conditions (Figure 5). NDRG1 phosphorylation decreased in SGK1-/-hearts a month after infarction (Figure 5A). This was accompanied by decreases in IκBα and NFκB2/p100 protein expression (Figure 5B) and increases in expression of all VEGF-A isoforms (Figure 5C). Thus, in pathological conditions, SGK1 ablation modulates similarly the signalling pathways observed in physiological conditions.

### Lack of SGK1 increases cardiac fibrosis and correlates with defective capillary formation after coronary artery ligation

**Figure 6:**
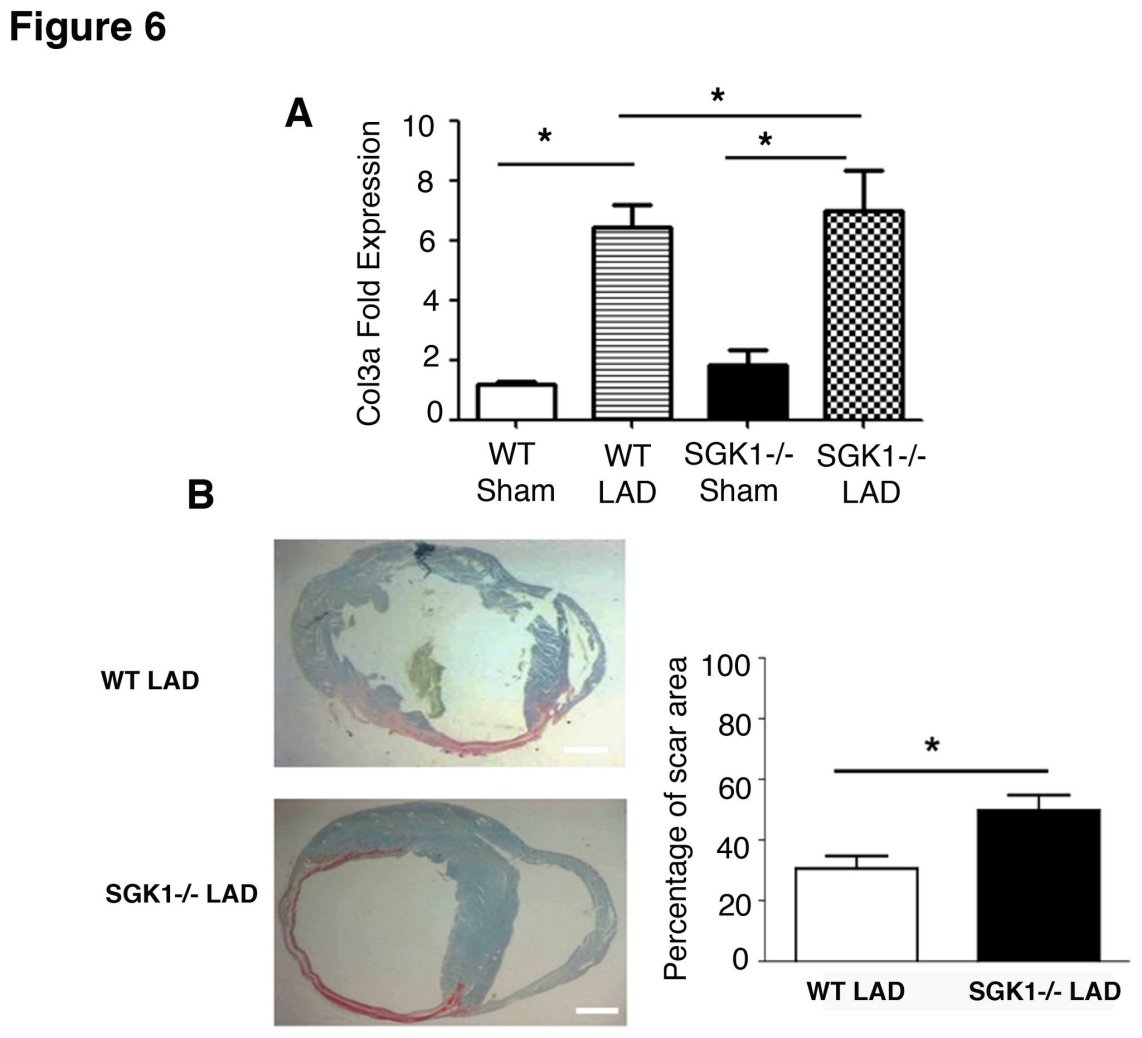
Effect of SGK1 ablation on cardiac collagen deposition after LAD injury. (A) Gene expression analysis of Col3a in WT and SGK1-/- heart one month after LAD. One-way ANOVA has been used to analyse significance among groups. * Indicates p<0.05 and n was ≥4 in each group. (B) Histological analysis of LAD operated hearts from WT and SGK1-/- mice one month after operation. Hearts were stained for the collagen marker, picro-sirius red. Epicardial and endocardial scar size was quantified in 3 different hearts in both WT and SGK1-/- and the mean value is the results of 10 distinct microscope field analysed in each group. Scale bars represent 1mm. Student t-test has been used to calculate p-value and * indicates p<0.05 (B). The data are reported as Mean ± SE.

Cardiac fibrosis was evaluated one month after myocardial ischemia in SGK1-/- and WT mice by histochemical analysis and collagen3a (Col3a) gene expression (Figure 6 A and B). Specifically, the expression level of Col3a was significantly increased after myocardial ischemia and was further upregulated in SGK1-/- mice (Figure 6A). Consistent with this genetic profile, immunohistochemical analysis of heart sections using the collagen marker Sirius red showed an increased collagen deposition and scar size in SGK1-/- mice (Figure 6B). 

**Figure 7:**
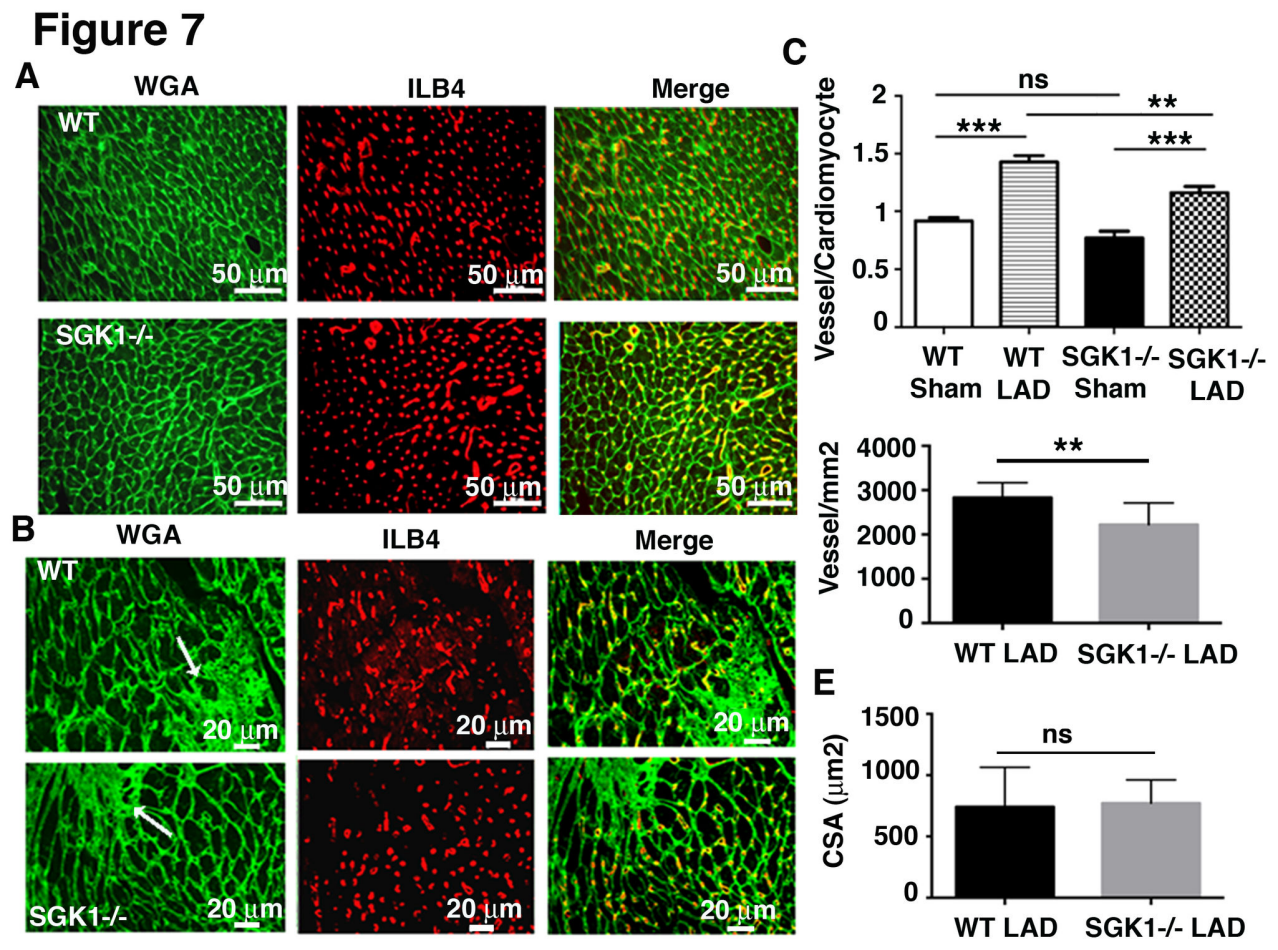
Reduced vessel density around the scar in SGK1-/- hearts. Microvessel density was measured in sham operated (A) and in LAD hearts (B). Microvessels were stained with biotinylated isolectin beta 4 (ILB4) and cardiomyocytes with WGA. Scale bars represent 50 µm in sham operated and 20 µm in LAD. The ratio of small vessels per cardiomyocytes (C) and per mm^2^ (D) is reported in the graphs. The number of hearts analysed was ≥4 in each group. One-way ANOVA was used to analyse significance among groups. (E) Measurements of cross-sectional area (CSA) 1 month after infarction in WT and KO mice. The number of hearts analysed was ≥4 in each group and ten distinct microscope fields for each slide were analysed. Ns, non-significant. ** Indicates p<0.01, *** p<0.001 and ns, non-significant. The data are reported as Mean ± SE.

To investigate the potential impact of defective ECs on cardiac recovery and neo-angiogenesis after ischemic insult, mice were analysed for vessel density. Isolectin B4 did not stain large vessels (Figure S5A) and was used to mark the microvasculature of WT and SGK1-/- hearts (Figure S5A and S5B). Vessel density was measured in heart sections before and after myocardial infarct (Figure 7 A and B) and was reported as number of ILB4-positive vessels per cardiomyocyte (Figure 7 C) and per mm^2^ (Figure 7D). No significant difference was observed between WT and SGK1-/- capillary density before myocardial ischemia (Figure 7A, C and D), however, one month after LAD the amount of capillary per cardiomyocyte and capillary per mm^2^ around the scar area was significantly lower in SGK1-/- hearts (Figure 7B, C and D). Measurements of cardiomyocyte cross-sectional area (Figure 7E) showed no significant differences between WT and SGK1-/-cardiomyocytes 1 month after myocardial infarct induction. The data suggests that decreased vessel formation may lead to worsening of cardiomyocyte mechanical stress or to increased cell death at specific time points after infarction.

## Discussion

The present study highlights a novel and pivotal role for SGK1 in adult cardiac repair after ischemic insult through promotion of neo-angiogenesis around the scar area. Analysis of the intracellular signalling pathways involved in SGK1 action revealed impaired NDRG1 phosphorylation in the heart, as well as in primary ECs, cardiomyocytes and fibroblasts of SGK1-/- mice. NDRG1 has been described as an important player in angiogenesis in different cell systems [27], although its role in cardiac tissue has not been explored in any detail. Indeed, both over-expression and phosphorylation of NDRG1 modified angiogenic gene expression in pancreatic cancer cells through down-regulation of the NF-κB pathway [9,10]. 

Here we demonstrated that decreased NDRG1 phosphorylation in SGK1-/- hearts was accompanied by down regulation of two NF-κB inhibitory components: IκBα and NF-κB_2_/p100 proteins. The NF-κB family consists of five members: NF-κB_1_ (p105/p50), NF-κB_2_ (p100/p52), RelA (p65), RelB and c-Rel [19]. In most cell types, the NF-κB complex is retained in the cytoplasm by inhibitors of κB proteins (IκBs). The C-terminal part of NFkB_2_/p100 also functions as an inhibitor, retaining the NF-κB complex in the cytoplasm [19]. IκBα and NF-κB_2_/p100 degradation leads to release of NF-κB DNA-binding proteins (p50 and p52) to the nucleus and results in transcription of its target genes [19]. To corroborate our data, proteomic analysis showed upregulation of the NF-κB activator Btk [25,26] in SGK1-/- hearts. These results indicate elevated NF-κB pathway activity in the heart in response to SGK1 ablation. 

Downregulation of both IκBα and NF-κB_2_/p100 in SGK1-/- hearts was accompanied by higher levels of VEGF-A, an NF-κB target protein in both physiological and pathological conditions. 

VEGF-A is a key regulatory component of physiological and pathological angiogenesis [28]. Notably, both under- and over-expression of VEGF-A disrupt normal development and vessel formation: deletion of VEGF-A caused embryonic lethality at day E9.5 [29], whereas overexpression of VEGF-A up to 2-3 fold resulted in severe cardiac development abnormality and embryonic lethality at day E12.5-E14 [30]. Mutant embryos over-expressing VEGF-A displayed cardiac malformation and aberrant coronary organization [30]. Similarly, in another study performed in quail embryos, injection of VEGF-A at day E4.5 caused cardiovascular malformation, enlarged heart and thin-walled myocardium [31]. Higher VEGF-A mRNA expression was also detected in SGK1-/- embryos at day E9.5 in the C57BL/6 mice background, which may in part explain their growth retardation, angiogenesis defect and embryonic lethality at day E10.5-E11.5 [3]. Accordingly, the smaller size of adult SGK1-/- mice in a 129/SvJ background can also be a result of higher VEGF-A protein level. Nevertheless, as SGK1 is a target of growth factor signalling pathways, the smaller phenotype could be a direct result of disrupted growth signalling in these mice. Indeed, we found that the phosphorylation level of ribosomal protein S6, implicated in protein synthesis signalling, was lowered in SGK1-/- mice (personal communication from the authors).

ECs are central players in angiogenesis and are targeted by VEGF-A. Primary ECs isolated from SGK1-/- hearts showed defective cell migration and failed to form organized networks when cultured on Matrigel. Importantly, re-expression of SGK1 by infection of an adenovirus encoding CA-SGK1 rescued formation of tube networks. Perturbation in VEGF-A signalling may explain the impaired behaviour of SGK1-/- ECs. Indeed, neutralizing VEGF-A in the media partially improved SGK1-/- EC tube formation capacity (Figure S2C) further implying a role for VEGF-A in this process. Although VEGF-A is a major player in angiogenesis, other signalling may contribute in synergy to enhance tissue revascularization. Angiogenesis occurs in several well characterized stages including degradation of vessel basement membrane, liberation, proliferation and migration of ECs to make appropriate connections [2], a defect in any of these steps may result in impaired tube formation and angiogenesis. Previous studies suggested a role for SGK1 and NDRG1 in cell migration through modification of adhesion molecules such as Vinculin and E-Cadherin [32], which may account for the lower capacity of endothelial cell migration in SGK1-/- mice leading to defective tube formation. 

Defective ECs impaired also cardiac remodelling and neo-angiogenesis *in vivo*, evident one month after coronary artery ligation. At this time, expression levels of fibrotic marker Col3a were significantly higher in SGK1-/- hearts, correlating with increased scar size and indicating an increase in the fibrotic response. Furthermore, the decrease in microvessel density around the scar area in SGK1-/- hearts clearly demonstrates a beneficial role for SGK1 in neo-angiogenesis and wound healing after myocardial ischemia. 

It is important to emphasise that other pathological models may lead to different outcome regarding the role of SGK1 in fibrosis and cardiac function. Some studies showed a profibrotic effect of SGK1 in response to mineralocorticoid deoxy-corticosterone acetate (DOCA) [33] and angiotensin-II *in vivo* [34]. Mineralocorticoids (DOCA) and angiotensin-II are known as potent inducers of high blood pressure leading to cardiac remodelling and hypertrophy [35,36]. The higher level of cardiac fibrosis in response to DOCA or angiotensin-II may appear to contradict the increased level of Col3a gene expression and scar formation in the absence of SGK1 after cardiac infarct. However, the cross-talk between SGK1 and the downstream signalling pathways activated in myocardial ischemia and hypertension, respectively, likely lead to disparate outcomes.

In summary, SGK1 is an important player in angiogenic processes of the heart after injury. Our analysis of perturbations in relevant downstream signalling pathways in its absence reveals new roles for both NDRG1 and NF-κB in cardiac pathology representing potential therapeutical and pharmacological targets in cardiac repair and regeneration studies. 

## Conclusion

Our collective findings establish a link between SGK1 and cardiac neo-angiogenesis, shedding new light on the signalling pathways implicated in physiologic and pathologic vessel formation.

## Supporting Information

Figure S1 **SGK2 and SGK3 protein expression in the WT and SGK1-/- hearts.** Western blot analysis of SGK2 and SGK3 protein expression in heart extracts from WT and SGK1-/- mice. GAPDH was used as loading control. Quantification of band intensities was performed using imageJ. N=4 in each group. Student t-test showed no significant (ns) differences between WT and SGK1-/-. The data are reported as Mean ± SE.
(TIF)

Figure S2 **VEGF expression in cardiac cells and tube formation analysis of ECs from WT and SGK1-/- hearts in response to neutralizing VEGF-A.** Quantification by Image J (see Material and Methods) of western blot analysis for the expression of VEGF-A proteins 20 and 25 in primary endothelial cells (A) and cardiomyocytes (B). GAPDH was used as loading control. The number of hearts used was ≥3 in each group. The data are reported as Mean ± SE. (C) Primary ECs from WT and SGK1-/- hearts were assessed for tube formation capacity in response to VEGF-A neutralizing antibody (2µg/ml). IgG was used as negative control. ECs were analysed for tube formation after 7h incubation with or without VEGF-A or IgG.
(TIF)

Figure S3 **Proteomic profile in SGK1-/- and WT hearts.** Three hearts from WT and SGK1-/- mice were lysed and proteins extracted in non-denaturing conditions by using lysis buffer from Kinexus. Fluorescent-labelled proteins were processed by Kinexus onto a Kinex^TM^ 850 antibody microarray chip. Fluorescent emission upon protein-antibody binding was detected and measured as Z-ratio (emission amount in SGK1-/- over WT). Expression and/or phosphorylation levels of different proteins were downregulated (negative values) or upregulated (positive values). Btk, regulator of NFkB activity, was upregulated in KO hearts confirming our western blot analyses.
(TIF)

Figure S4 **Re-expression of SGK1 rescued tube network formation in KO ECs.** ECs from SGK1-/- hearts were isolated and infected with an adenovirus expressing the constitutive active form of SGK1 (Ad-SGK1 CA-GFP) and with a control adenovirus expressing beta-galactosidase (βGal; Ad βGal-GFP). Both viruses co-expressed green fluorescent protein (GFP), as shown in panels (A) 12 hours after viral infection. Infected SGK1-/- ECs formed tube networks 7 hours after plating in matrigel, conversely to ECs infected with control virus (B). Total cell extracts from Ad-SGK1 CA-GFP and Ad βGal-GFP infected ECs were analysed for SGK1 expression. Blots were normalized with alpha-tubulin. Arrow indicates SGK1 expression in KO ECs infected with Ad-SGK1 CA-GFP or control virus (C). The reported data have been assessed in two independent experiments. In each independent experiment, three separate analyses were performed.
(TIF)

Figure S5 **Isolectin beta 4 staining of vessels.** Microvessels were stained in WT (A) and SGK1-/- hearts (B) with biotinylated isolectin beta 4 (ILB4). Nuclei were visualized with DAPI staining. Scale bars represent 50µm and arrows point to small vessels.
(TIF)

## References

[1] Carmeliet P (2000) Mechanisms of angiogenesis and arteriogenesis. Nat Med 6: 389-395. doi:10.1038/74651. PubMed: 10742145. 10742145

[2] Carmeliet P, Jain RK (2011) Molecular mechanisms and clinical applications of angiogenesis. Nature 473: 298-307. doi:10.1038/nature10144. PubMed: 21593862. 21593862 PMC4049445

[3] Catela C, Kratsios P, Hede M, Lang F, Rosenthal N (2010) Serum and glucocorticoid-inducible kinase 1 (SGK1) is necessary for vascular remodeling during angiogenesis. Dev Dyn 239: 2149-2160. doi:10.1002/dvdy.22345. PubMed: 20568246. 20568246

[4] Webster MK, Goya L, Ge Y, Maiyar AC, Firestone GL (1993) Characterization of sgk, a novel member of the serine/threonine protein kinase gene family which is transcriptionally induced by glucocorticoids and serum. Mol Cell Biol 13: 2031-2040. PubMed: 8455596. 8455596 10.1128/mcb.13.4.2031-2040.1993 PMC359524

[5] Park J, Leong ML, Buse P, Maiyar AC, Firestone GL et al. (1999) Serum and glucocorticoid-inducible kinase (SGK) is a target of the PI 3-kinase-stimulated signaling pathway. EMBO J 18: 3024-3033. doi:10.1093/emboj/18.11.3024. PubMed: 10357815. 10357815 PMC1171384

[6] Tessier M, Woodgett JR (2006) Serum and glucocorticoid-regulated protein kinases: variations on a theme. J Cell Biochem 98: 1391-1407. doi:10.1002/jcb.20894. PubMed: 16619268. 16619268

[7] Murray JT, Campbell DG, Morrice N, Auld GC, Shpiro N et al. (2004) Exploitation of KESTREL to identify NDRG family members as physiological substrates for SGK1 and GSK3. Biochem J 384: 477-488. doi:10.1042/BJ20041057. PubMed: 15461589. 15461589 PMC1134133

[8] Kovacevic Z, Richardson DR (2006) The metastasis suppressor, Ndrg-1: a new ally in the fight against cancer. Carcinogenesis 27: 2355-2366. doi:10.1093/carcin/bgl146. PubMed: 16920733. 16920733

[9] Murakami Y, Hosoi F, Izumi H, Maruyama Y, Ureshino H et al. (2010) Identification of sites subjected to serine/threonine phosphorylation by SGK1 affecting N-myc downstream-regulated gene 1 (NDRG1)/Cap43-dependent suppression of angiogenic CXC chemokine expression in human pancreatic cancer cells. Biochem Biophys Res Commun 396: 376-381. doi:10.1016/j.bbrc.2010.04.100. PubMed: 20416281. 20416281

[10] Hosoi F, Izumi H, Kawahara A, Murakami Y, Kinoshita H et al. (2009) N-myc downstream regulated gene 1/Cap43 suppresses tumor growth and angiogenesis of pancreatic cancer through attenuation of inhibitor of kappaB kinase beta expression. Cancer Res 69: 4983-4991. doi:10.1158/0008-5472.CAN-08-4882. PubMed: 19491262. 19491262

[11] Wulff P, Vallon V, Huang DY, Völkl H, Yu F et al. (2002) Impaired renal Na(+) retention in the sgk1-knockout mouse. J Clin Invest 110: 1263-1268. doi:10.1172/JCI0215696. PubMed: 12417564. 12417564 PMC151609

[12] Santini MP, Tsao L, Monassier L, Theodoropoulos C, Carter J et al. (2007) Enhancing repair of the mammalian heart. Circ Res 100: 1732-1740. doi:10.1161/CIRCRESAHA.107.148791. PubMed: 17525368. 17525368 PMC3227120

[13] Sakamoto K, Zarrinpashneh E, Budas GR, Pouleur AC, Dutta A et al. (2006) Deficiency of LKB1 in heart prevents ischemia-mediated activation of AMPKalpha2 but not AMPKalpha1. Am J Physiol Endocrinol Metab 290: E780-E788. doi:10.1152/ajpendo.00443.2005. PubMed: 16332922. 16332922 PMC2128705

[14] Lara-Pezzi E, Winn N, Paul A, McCullagh K, Slominsky E et al. (2007) A naturally occurring calcineurin variant inhibits FoxO activity and enhances skeletal muscle regeneration. J Cell Biol 179: 1205-1218. doi:10.1083/jcb.200704179. PubMed: 18086917. 18086917 PMC2140042

[15] Siedlecka U, Arora M, Kolettis T, Soppa GK, Lee J et al. (2008) Effects of clenbuterol on contractility and Ca2+ homeostasis of isolated rat ventricular myocytes. Am J Physiol Heart Circ Physiol 295: H1917-H1926. doi:10.1152/ajpheart.00258.2008. PubMed: 18775853. 18775853 PMC2614565

[16] Ibrahim M, Al Masri A, Navaratnarajah M, Siedlecka U, Soppa GK et al. (2010) Prolonged mechanical unloading affects cardiomyocyte excitation-contraction coupling, transverse-tubule structure, and the cell surface. FASEB J 24: 3321-3329. doi:10.1096/fj.10-156638. PubMed: 20430793. 20430793 PMC2923356

[17] Kanda S, Miyata Y, Kanetake H (2004) Fibroblast growth factor-2-mediated capillary morphogenesis of endothelial cells requires signals via Flt-1/vascular endothelial growth factor receptor-1: possible involvement of c-Akt. J Biol Chem 279: 4007-4016. PubMed: 14610089. 14610089 10.1074/jbc.M307569200

[18] Aoyama T, Matsui T, Novikov M, Park J, Hemmings B et al. (2005) Serum and glucocorticoid-responsive kinase-1 regulates cardiomyocyte survival and hypertrophic response. Circulation 111: 1652-1659. doi:10.1161/01.CIR.0000160352.58142.06. PubMed: 15795328. 15795328

[19] Bonizzi G, Karin M (2004) The two NF-kappaB activation pathways and their role in innate and adaptive immunity. Trends Immunol 25: 280-288. doi:10.1016/j.it.2004.03.008. PubMed: 15145317. 15145317

[20] Djordjević G, Matusan-Ilijas K, Sinozić E, Damante G, Fabbro D et al. (2008) Relationship between vascular endothelial growth factor and nuclear factor-kappaB in renal cell tumors. Croat Med J 49: 608-617. doi:10.3325/cmj.2008.5.608. PubMed: 18925694. 18925694 PMC2582353

[21] Fu J, Bian M, Jiang Q, Zhang C (2007) Roles of Aurora kinases in mitosis and tumorigenesis. Mol Cancer Res 5: 1-10. doi:10.1158/1541-7786.MCR-06-0208. PubMed: 17259342. 17259342

[22] Peterson QP, Goode DR, West DC, Ramsey KN, Lee JJ et al. (2009) PAC-1 activates procaspase-3 in vitro through relief of zinc-mediated inhibition. J Mol Biol 388: 144-158. doi:10.1016/j.jmb.2009.03.003. PubMed: 19281821. 19281821 PMC2714579

[23] Rohan PJ, Davis P, Moskaluk CA, Kearns M, Krutzsch H et al. (1993) PAC-1: a mitogen-induced nuclear protein tyrosine phosphatase. Science 259: 1763-1766. doi:10.1126/science.7681221. PubMed: 7681221. 7681221

[24] Lolli G, Lowe ED, Brown NR, Johnson LN (2004) The crystal structure of human CDK7 and its protein recognition properties. Structure 12: 2067-2079. doi:10.1016/j.str.2004.08.013. PubMed: 15530371. 15530371

[25] Doyle SL, Jefferies CA, O'Neill LA (2005) Bruton's tyrosine kinase is involved in p65-mediated transactivation and phosphorylation of p65 on serine 536 during NFkappaB activation by lipopolysaccharide. J Biol Chem 280: 23496-23501. doi:10.1074/jbc.C500053200. PubMed: 15849198. 15849198

[26] Shinners NP, Carlesso G, Castro I, Hoek KL, Corn RA et al. (2007) Bruton's tyrosine kinase mediates NF-kappa B activation and B cell survival by B cell-activating factor receptor of the TNF-R family. J Immunol 179: 3872-3880. PubMed: 17785824. 17785824 10.4049/jimmunol.179.6.3872

[27] Bandyopadhyay S, Pai SK, Gross SC, Hirota S, Hosobe S et al. (2003) The Drg-1 gene suppresses tumor metastasis in prostate cancer. Cancer Res 63: 1731-1736. PubMed: 12702552. 12702552

[28] Nowak DG, Amin EM, Rennel ES, Hoareau-Aveilla C, Gammons M et al. (2010) Regulation of vascular endothelial growth factor (VEGF) splicing from pro-angiogenic to anti-angiogenic isoforms: a novel therapeutic strategy for angiogenesis. J Biol Chem 285: 5532-5540. doi:10.1074/jbc.M109.074930. PubMed: 19906640. 19906640 PMC2820781

[29] Carmeliet P, Ferreira V, Breier G, Pollefeyt S, Kieckens L et al. (1996) Abnormal blood vessel development and lethality in embryos lacking a single VEGF allele. Nature 380: 435-439. doi:10.1038/380435a0. PubMed: 8602241. 8602241

[30] Miquerol L, Langille BL, Nagy A (2000) Embryonic development is disrupted by modest increases in vascular endothelial growth factor gene expression. Development 127: 3941-3946. PubMed: 10952892. 10952892 10.1242/dev.127.18.3941

[31] Feucht M, Christ B, Wilting J (1997) VEGF induces cardiovascular malformation and embryonic lethality. Am J Pathol 151: 1407-1416. PubMed: 9358767. 9358767 PMC1858086

[32] Schmidt EM, Gu S, Anagnostopoulou V, Alevizopoulos K, Föller M et al. (2012) Serum- and glucocorticoid-dependent kinase-1-induced cell migration is dependent on vinculin and regulated by the membrane androgen receptor. FEBS J 279: 1231-1242. doi:10.1111/j.1742-4658.2012.08515.x. PubMed: 22309306. 22309306

[33] Vallon V, Wyatt AW, Klingel K, Huang DY, Hussain A et al. (2006) SGK1-dependent cardiac CTGF formation and fibrosis following DOCA treatment. J Mol Med (Berl) 84: 396-404. doi:10.1007/s00109-005-0027-z. PubMed: 16604333. 16604333

[34] Yang M, Zheng J, Miao Y, Wang Y, Cui W et al. (2012) Serum/Glucocorticoid-Regulated Kinase 1 Regulates Alternatively Activated Macrophage Polarization Contributing to Angiotensin II-Induced Inflammation and Cardiac Fibrosis. Arterioscler Thromb Vasc Biol. 10.1161/ATVBAHA.112.248732 22556335

[35] Karam H, Heudes D, Gonzales MF, Löffler BM, Clozel M et al. (1996) Respective role of humoral factors and blood pressure in aortic remodeling of DOCA hypertensive rats. Am J Hypertens 9: 991-998. doi:10.1016/0895-7061(96)00119-7. PubMed: 8896651. 8896651

[36] Zimmerman D, Burns KD (2012) Angiotensin-(1-7) in kidney disease: a review of the controversies. Clin Sci (Lond) 123: 333-346. doi:10.1042/CS20120111. PubMed: 22639821. 22639821

