# Supplementary figures and images for: Ablation of SGK1 Impairs Endothelial Cell Migration and Tube Formation Leading to Decreased Neo-Angiogenesis Following Myocardial Infarction
Source: PLoS One. 2013 Nov 12;8(11):e80268. doi: 10.1371/journal.pone.0080268 (PMC3827188; doi:10.1371/journal.pone.0080268)

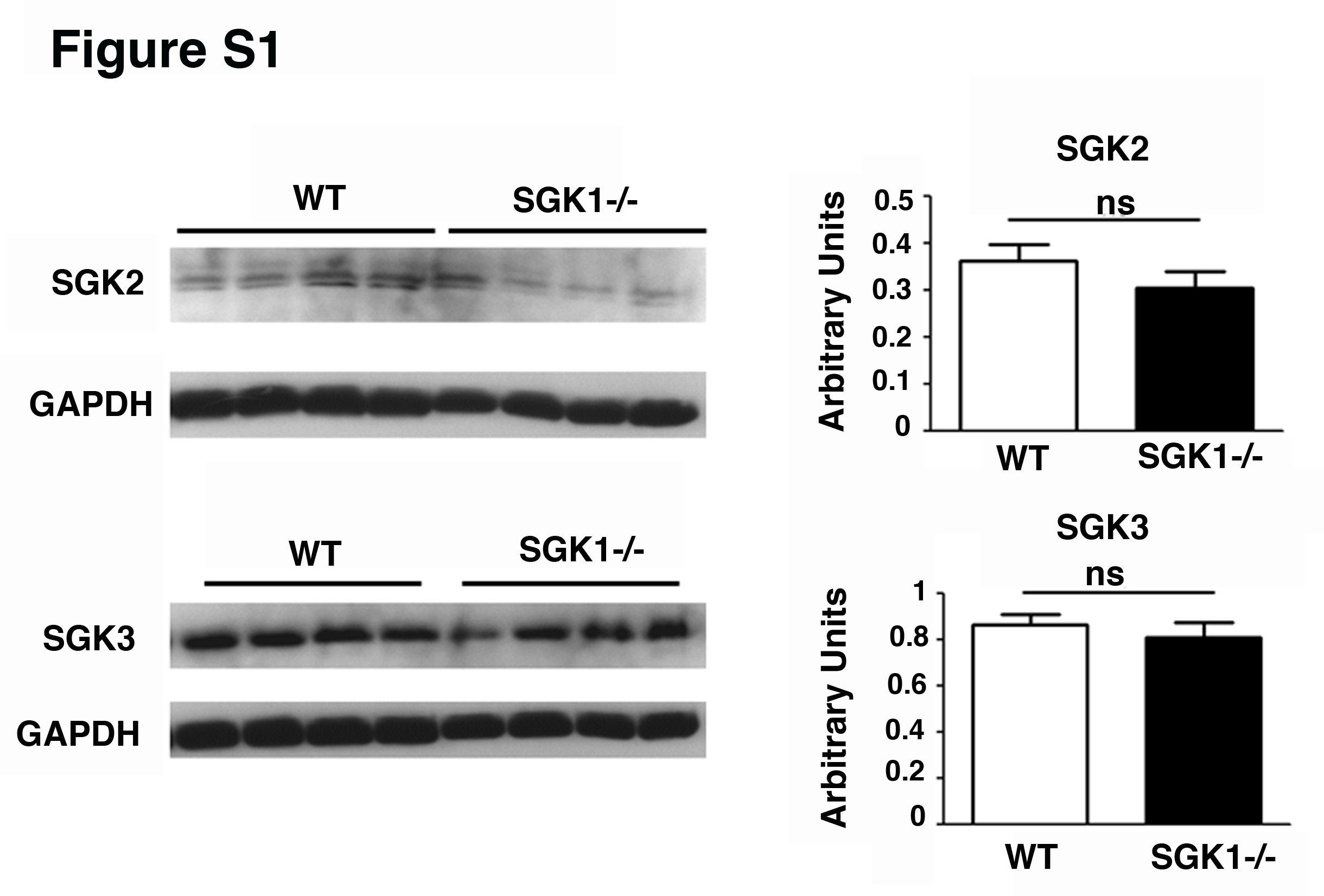

Supplement: Figure S1 — SGK2 and SGK3 protein expression in the WT and SGK1-/- hearts. Western blot analysis of SGK2 and SGK3 protein expression in heart extracts from WT and SGK1-/- mice. GAPDH was used as loading control. Quantification of band intensities was performed using imageJ. N=4 in each group. Student t-test showed no significant (ns) differences between WT and SGK1-/-. The data are reported as Mean ± SE. (TIF) [file pone.0080268.s001.tif]

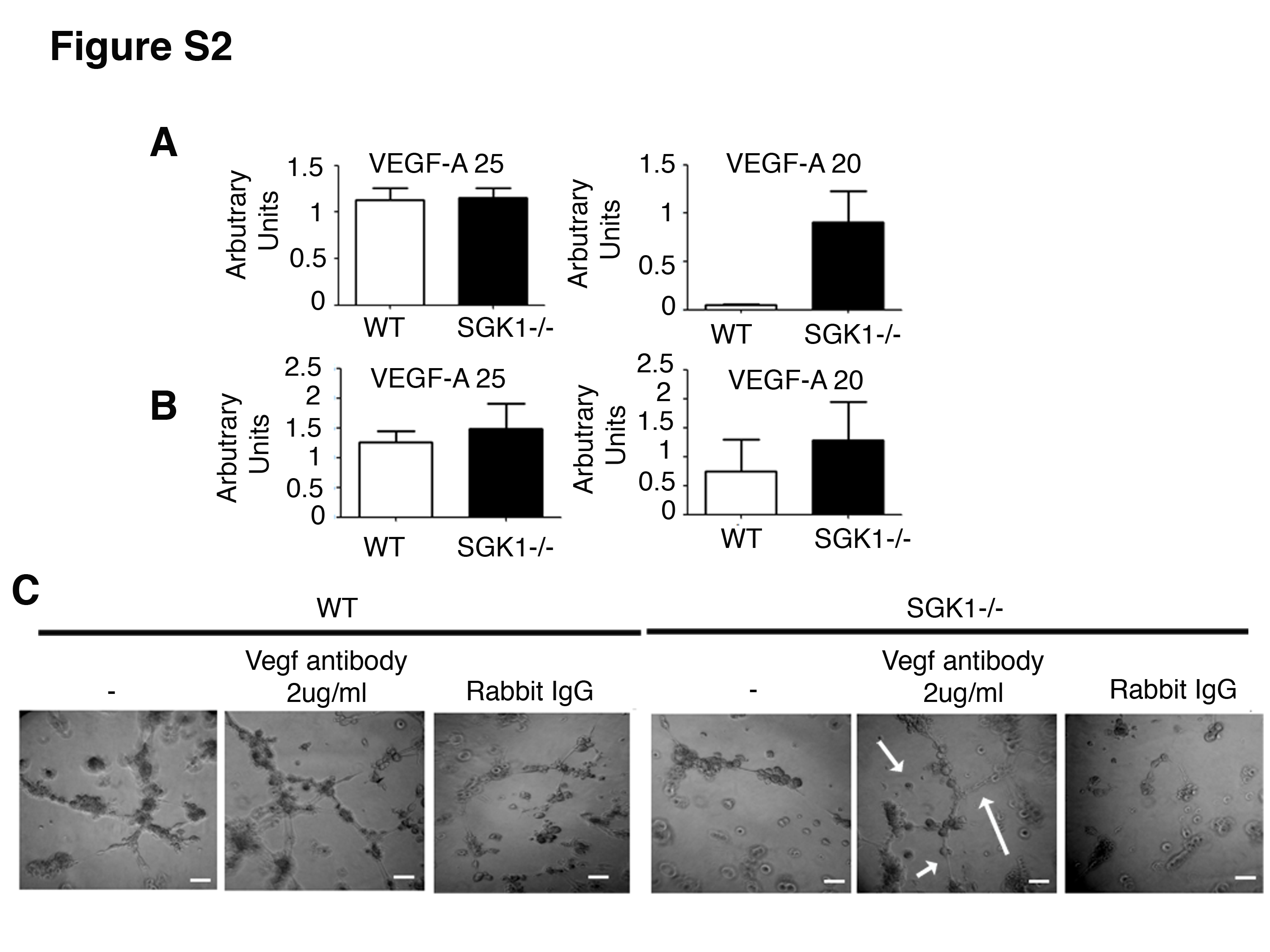

Supplement: Figure S2 — VEGF expression in cardiac cells and tube formation analysis of ECs from WT and SGK1-/- hearts in response to neutralizing VEGF-A. Quantification by Image J (see Material and Methods) of western blot analysis for the expression of VEGF-A proteins 20 and 25 in primary endothelial cells (A) and cardiomyocytes (B). GAPDH was used as loading control. The number of hearts used was ≥3 in each group. The data are reported as Mean ± SE. (C) Primary ECs from WT and SGK1-/- hearts were assessed for tube formation capacity in response to VEGF-A neutralizing antibody (2µg/ml). IgG was used as negative control. ECs were analysed for tube formation after 7h incubation with or without VEGF-A or IgG. (TIF) [file pone.0080268.s002.tif]

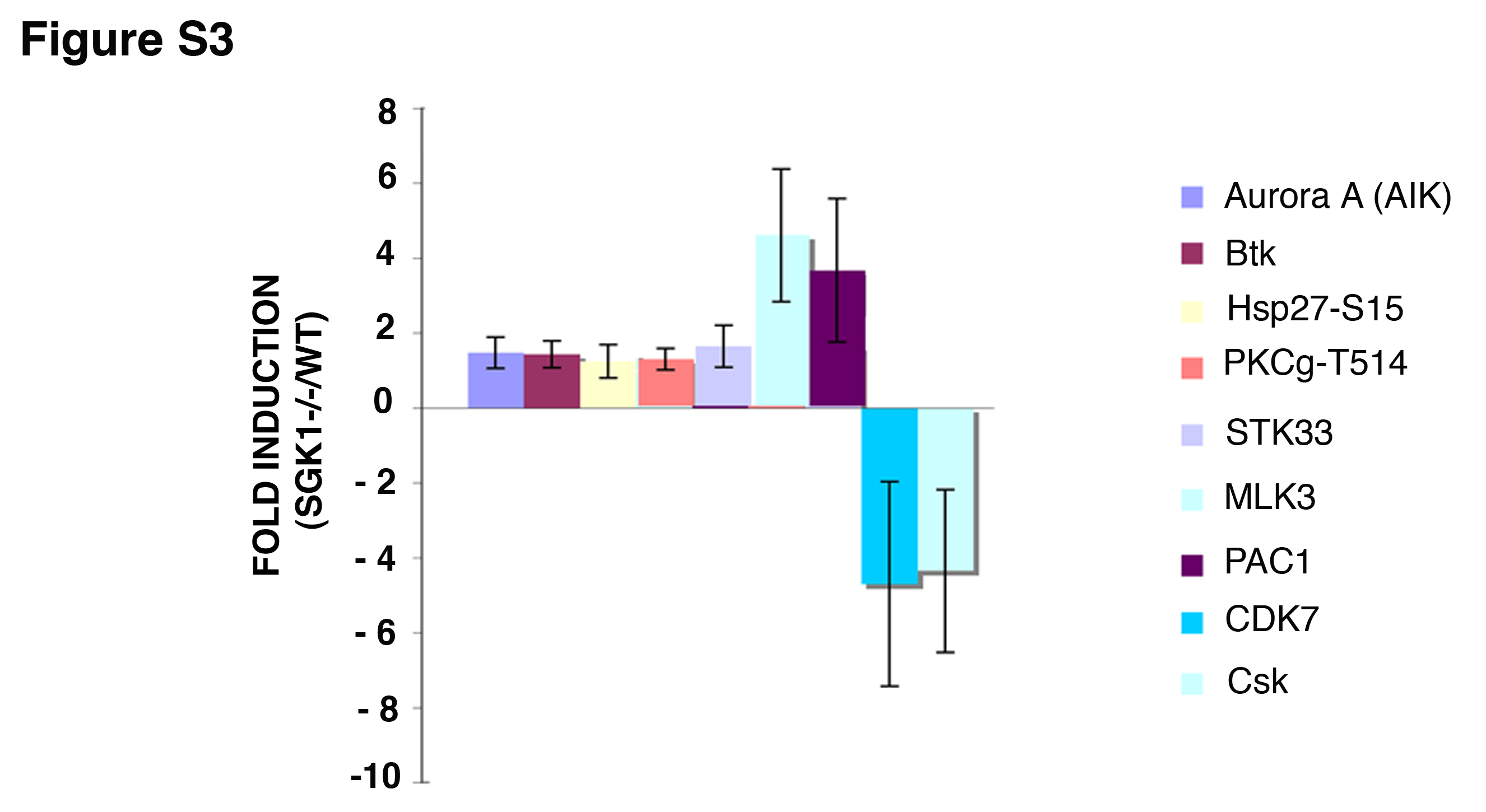

Supplement: Figure S3 — Proteomic profile in SGK1-/- and WT hearts. Three hearts from WT and SGK1-/- mice were lysed and proteins extracted in non-denaturing conditions by using lysis buffer from Kinexus. Fluorescent-labelled proteins were processed by Kinexus onto a KinexTM 850 antibody microarray chip. Fluorescent emission upon protein-antibody binding was detected and measured as Z-ratio (emission amount in SGK1-/- over WT). Expression and/or phosphorylation levels of different proteins were downregulated (negative values) or upregulated (positive values). Btk, regulator of NFkB activity, was upregulated in KO hearts confirming our western blot analyses. (TIF) [file pone.0080268.s003.tif]

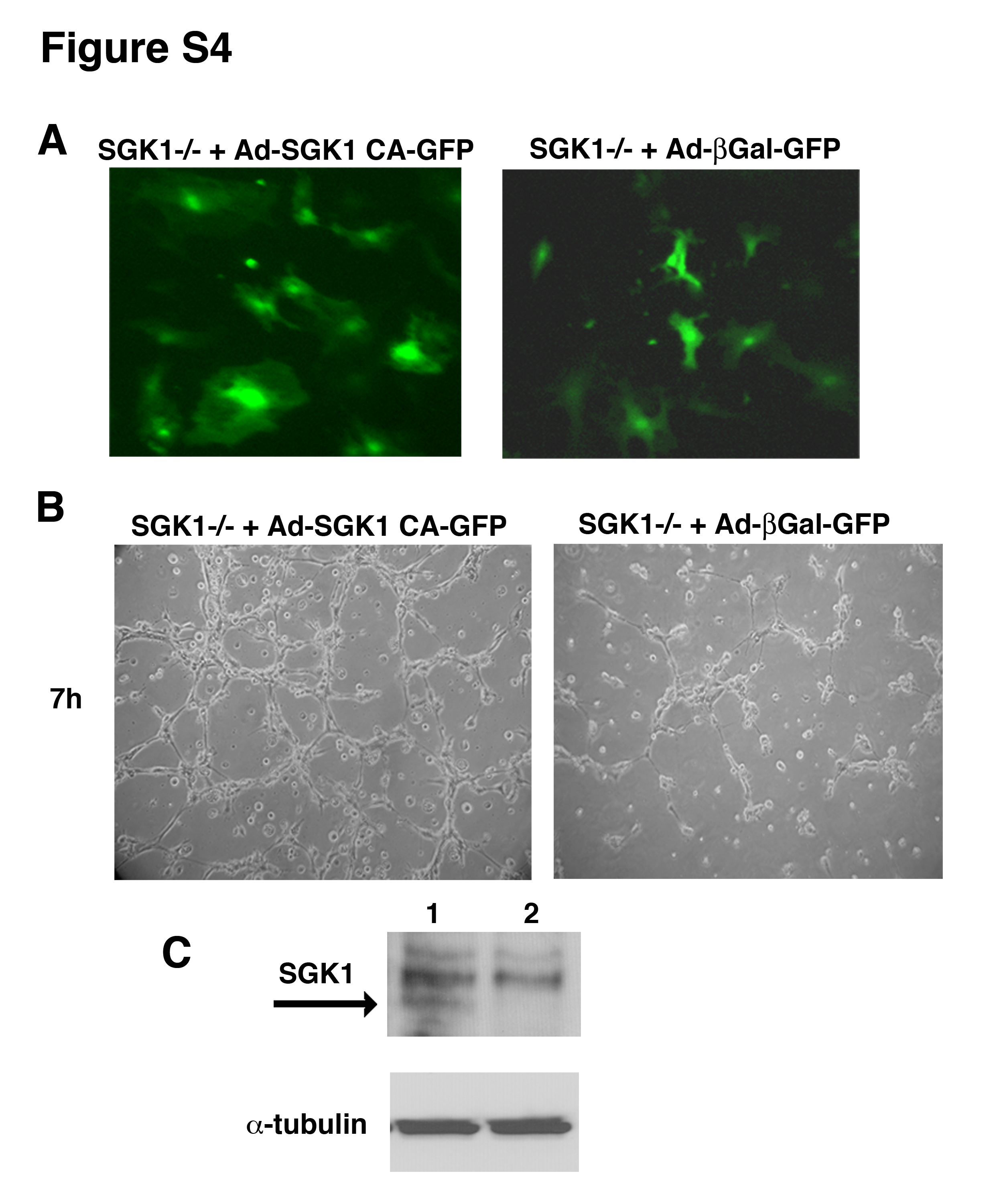

Supplement: Figure S4 — Re-expression of SGK1 rescued tube network formation in KO ECs. ECs from SGK1-/- hearts were isolated and infected with an adenovirus expressing the constitutive active form of SGK1 (Ad-SGK1 CA-GFP) and with a control adenovirus expressing beta-galactosidase (βGal; Ad βGal-GFP). Both viruses co-expressed green fluorescent protein (GFP), as shown in panels (A) 12 hours after viral infection. Infected SGK1-/- ECs formed tube networks 7 hours after plating in matrigel, conversely to ECs infected with control virus (B). Total cell extracts from Ad-SGK1 CA-GFP and Ad βGal-GFP infected ECs were analysed for SGK1 expression. Blots were normalized with alpha-tubulin. Arrow indicates SGK1 expression in KO ECs infected with Ad-SGK1 CA-GFP or control virus (C). The reported data have been assessed in two independent experiments. In each independent experiment, three separate analyses were performed. (TIF) [file pone.0080268.s004.tif]

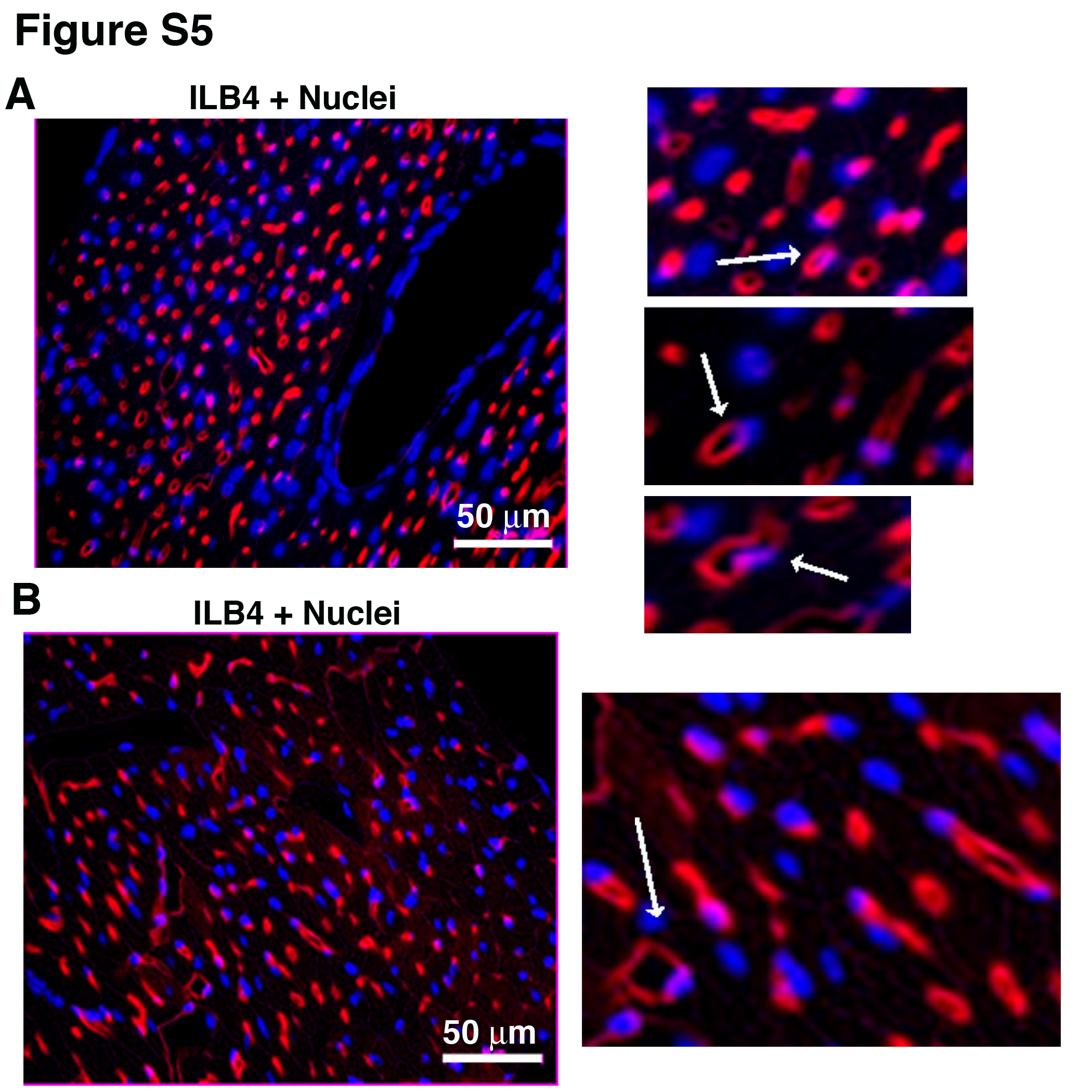

Supplement: Figure S5 — Isolectin beta 4 staining of vessels. Microvessels were stained in WT (A) and SGK1-/- hearts (B) with biotinylated isolectin beta 4 (ILB4). Nuclei were visualized with DAPI staining. Scale bars represent 50µm and arrows point to small vessels. (TIF) [file pone.0080268.s005.tif]
